# Supplementary material for: The Distribution of Furfuryl Alcohol (FA) Resin in Bamboo Materials after Surface Furfurylation
Source: Materials (Basel). 2020 Mar 5;13(5):1157. doi: 10.3390/ma13051157 (PMC7084994; doi:10.3390/ma13051157)
Supplement: Supplementary file 1 [file materials-13-01157-s001.pdf]

materials

Supplementary

The Furfuryl Alcohol (FA) Resin Distribution in Surface Furfurylation Bamboo

Minghui Liu [1,2,†], Wanju Li [3,†], Hankun Wang [1,2,*], Xuexia Zhang [4] and Yan Yu [4,*]

[1] Institute of New Bamboo and Rattan Based Biomaterials, International Center for Bamboo and Rattan, Beijing, 100102, China; lmh1519@163.com
[2] SFA and Beijing Co-built Key Lab for Bamboo and Rattan Science & Technology, Beijing, 100102, China
[3] Guangdong Provincial Key Laboratory of Silviculture, Protection and Utilization, Guangdong Academy of Forestry, Guangzhou, 510520, China; liwanju2011@126.com
[4] College of Material Engineering, Fujian Agriculture and Forestry University, Fuzhou, 350002, China; 13121417614@163.com
* Correspondence: wanghankun@icbr.ac.cn (H.W.); yuyan9812@outlook.com (Y.Y.)
† These authors contributed equally to this work.

Received: 05 February 2020; Accepted: 3 March 2020; Published: 3 March 2020

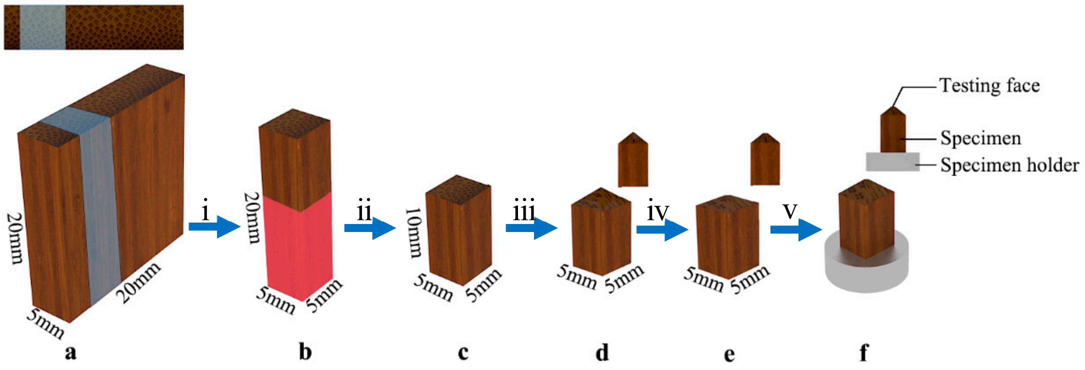

Figure S1. The specimen preparation procedure for nanoindentation: (i) the sample was swan up according to the selected area form (a) to (b); (ii) the sample was swan up into the sample based on the selected area from (b) to (c); (iii) cutting the upper surface of the specimen into a pyramid shape; (iv) cutting the top of the pyramid to obtain a suitable and clean test surface; (v) the sample was mounted on a custom iron sample holder with epoxy resin glue.

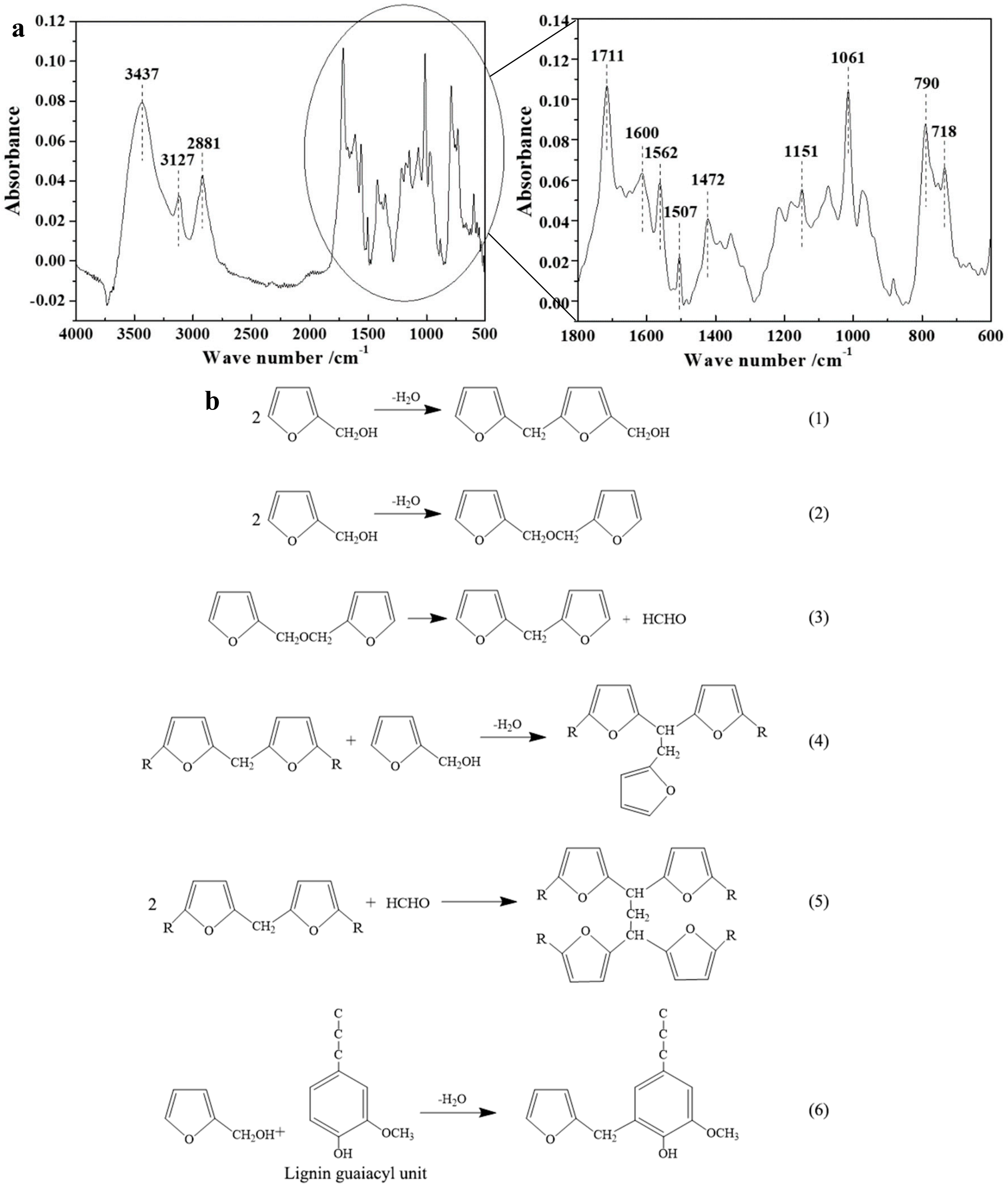

Figure S2. Characterization of FA resin and reaction of FA under acidic conditions: (a) FTIR spectra; (b) The possible reaction during furfurylation process: (1) condensation reaction forming a dimer of FA; (2) condensation reaction, termination of polymerization; (3) loss of formaldehyde during the reaction; (4) and (5) cross-linking patterns of furfuryl alcohol-polymer chains; (6) suggested grafting reaction between FA and a guaiacyl unit of lignin.

© 2020 by the authors. Licensee MDPI, Basel, Switzerland. This article is an open access article distributed under the terms and conditions of the Creative Commons Attribution (CC BY) license (http://creativecommons.org/licenses/by/4.0/).
